# The anticaries effects of pit and fissure sealant in the first permanent molars of school-age children from Guangzhou: a population-based cohort study

**DOI:** 10.1186/s12903-019-0846-x

**Published:** 2019-07-16

**Authors:** Weijia Liu, Lihua Xiong, Jianbo Li, Chongshan Guo, Weihua Fan, Shaohong Huang

**Affiliations:** 1 Faculty of School Health, Guangzhou Centre for Disease Control and Prevention, Guangzhou, China; 2 Stomatological Hospital, Southern Medical University, No. 366, south of Jiangnan Avenue, Guangzhou, People’s Republic of China

**Keywords:** Pit and fissure sealant, Dental caries, Cumulative incidence of caries

## Abstract

**Background:**

Analyses of the effects of pit and fissure sealant have been based on small samples and lack large-scale field evaluation data in China. The aim of this study was to understand the effect of pit and fissure sealant in preventing caries in the first permanent molars (FPMs) of children in Guangzhou.

**Methods:**

We conducted a population-based cohort study using the database of the pit and fissure sealant program of Guangzhou. The carious status and sealant retention of the FPMs were assessed in 4,822 school children who received pit and fissure sealant 3 years prior to the study. The control group included 4,396 children who had indications for receiving pit and fissure sealant but were not treated and were matched according to sex, age and school.

**Results:**

In the sealant group, the rate of sealant retention in the FPMs was 72.2%. Children in the sealant group had a 37% decreased risk of dental caries compared with the control group (adjusted HR = 0.63 [95% confidence interval (CI), 0.57–0.69], *P* < 0.001). Compared to no sealant use, the use of pit and fissure sealants reduced the risk of developing dental caries by 44% after 3 years in the FPMs of children from rural areas, reflecting a greater reduction than that among urban children (35%) during the same period (urban: adjusted hazard ratio (HR) = 0.65 [95% CI, 0.58–0.72]; rural: adjusted HR = 0.56 [95% CI, 0.45–0.70], *P* < 0.001). The mean number of decayed, missing, or filled permanent teeth (DMFT) in the control group was higher than that in the sealant group, and the difference was statistically significant regardless of sex.

**Conclusions:**

Pit and fissure sealant has a significant preventive effect against dental caries in the FPMs, especially for children in rural areas; thus, this sealant represents an effective technique for preventing and controlling dental caries.

## Background

Dental caries, a highly prevalent oral disease in children, occurs frequently in the first permanent molars [1–3], and the prevalence of the disease is closely linked to social and economic disadvantages [4, 5]. For example, the National Survey 2011–2012 in the United States showed that nearly one-fourth of children and more than one-half of adolescents suffer from dental carious lesions in their permanent teeth [6]. Furthermore, a report from the American Dental Association Council on Scientific Affairs indicated that more caries were preset in the pits and fissures of teeth than in the smooth surfaces in the adolescent population [7]. The pit and fissure sealant technique is a very effective method for reducing the occurrence of pit and fissure caries; thus, it is currently used extensively in developed countries and developing countries [8–11].

Data from the 2009 survey of children in Guangzhou indicated that 2.9% of children aged 6 years had caries in permanent teeth while 60.8% of these children had caries in primary teeth, 17.3% of children aged 9 years had caries in permanent teeth while 64.9% had caries in primary teeth, and 29.4% of children aged 12 years had caries in permanent teeth. Furthermore, pits and fissures of the molars, especially the first permanent molar (FPMs), are most vulnerable to dental caries, and approximately 60% of caries in the FPMs and approximately 90% of caries in permanent teeth occur in pits and fissures [12, 13].

To reduce the incidence of dental caries in children, a major public health project providing free pit and fissure sealant for the FPMs of school-age children was implemented in 2011. Assessing the anticaries effect of this major public health project and providing a scientific basis for government decision-making are important tasks. Furthermore, such assessments can also provide a reference for the development of pit and fissure sealant projects in other cities. However, to our knowledge, analyses of the effects of pit and fissure sealant have been based on small samples and lack large-scale field evaluation data in China. Therefore, the purpose of our study was to assess the effectiveness of a population-based FPM pit and fissure sealant program for Chinese school-age children with 3 years of follow-up. The secondary objective was to assess sealant retention.

## Methods

### Study setting

The data source was the database of the Guangzhou program providing free pit and fissure sealant for the FPMs of school-age children. This project included all grade 2 primary school children aged 6–8 years from 12 districts of Guangzhou City. The project included oral health examinations and free pit and fissure sealant. Approximately 120,000 children have participated in this project, covering more children and teeth than projects in any other city [14].

The Guangzhou program with free pit and fissure sealant was conducted under a team composed of public health officials, professionals in the Guangzhou Center for Disease Control and Prevention, dental advisors and dentists. Meanwhile, a series of training sessions was conducted to improve skills among all program personnel. The topics included the program implementation guide, including the standardized criteria for screening high-caries risk children and teeth, the technique for sealant application, standard infection control procedures, data collection methods and so on. Informed consent was obtained from all 2nd grade students’ parents to allow their children to participate in the project.

All 2nd grade students were screened by practitioners (who were required have a practicing doctor’s or assistant practicing doctor’s qualifications) in schools and were later treated with fissure sealants in clinic services if indicated (the inclusion criteria required the child’s and the parent’s or guardian’s consent and the presence of deep pits and fissures in the FPMs. Children were excluded when a dentinal carious lesion was present on one of the FPMs or the FPMs were completely or partially unerupted). Of the 120,000 enrolled children, 68,000 children had indications for pit and fissure sealant on the FPMs, and 26,000 children had received pit and fissure sealant on the FPMs. In this study, we applied resin-based sealants (3 M ESPE Concise Light Cured White Sealant 6 ml, USA and etc.).

### Study participants

**Fig. 1:**
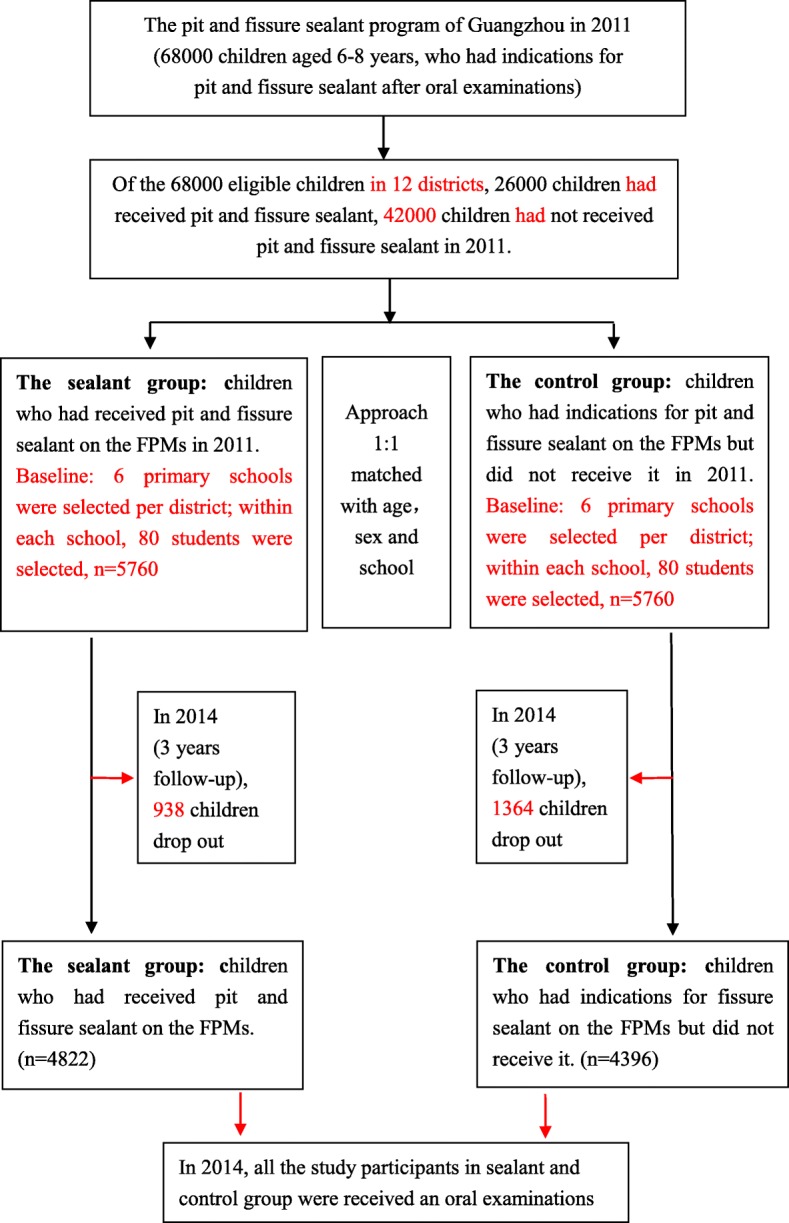
Participant selection flowchart

This was a population-base cohort study that used the database of pit and fissure sealant program of Guangzhou. To create the study cohort, the children who had received pit and fissure sealant on the FPMs in September 2011 were selected as the sealant group, and children who had indications for fissure sealant but did not receive the sealant served as the control group and were matched according to sex, age and school. Figure 1 shows a study flowchart for the selection process.

### Sample size calculation

In each district of Guangzhou, the sample size calculation was based on an estimated 30% caries incidence in the control group, the highest odds ratio of 0.6 at 3 years of follow-up, a correlation of 0.1 for the outcome (the incidence of caries) between the sealant group and control teeth, an alpha level of 0.05 and a power of 0.9 [15]. Based on these values, a sample size of approximately 400 school students was determined. Considering some factors such as loss to follow-up, a total of 480 school students were randomly chosen from the selected primary school in each district of Guangzhou. To evaluate the dental caries prevention effect of pit and fissure sealant after 3 years, 480 students were selected for the sealant group and for the control group. To acquire results for the entire city, in this study, we combine data from 12 districts of Guangzhou city.

A multi-stage stratified random sampling method was used to obtain a representative sample in each district. First, using a random number generator, 6 primary schools in each administrative region were selected using sampling. Within each primary school, 80 children who had received pit and fissure sealant 3 years previously were selected for inclusion in the sealant group. Meanwhile, the control group included students in the same grade who had indications for pit and fissure sealant but did not accept the sealant treatment.

After the follow-up period ended in September 2014, we conducted oral examinations among the study participants. In the sealant group, the dentists assessed the retention of the sealant and the presence of dental caries. In the control group, the dentists checked for dental caries in the FPMs that had an indication for pit and fissure sealant but did not receive it. A nurse was responsible for preparing surgical instruments and completing the written record. The professionals at the Guangzhou Center for Disease Control and Prevention were responsible for inspecting the site’s organization, coordination and logistic support. According to the criteria for clinical dentition examination of the third national oral health survey [16], dental caries in the FPMs were recorded using a community periodontal index (CPI) probe. The investigation team consisted of five oral practitioners, five nurses and two professionals at the Guangzhou Center for Disease Control and Prevention. Prior to the assessment, technical experts from the technical guidance group of Guangzhou provided technical training for on-site dental staff to unify the standards used and asses the consistency of this project. A senior dentist and nurse on the investigation team were responsible for quality control at the scene. During the investigation period, a consistency test was conducted in each district. The kappa value of the consistency test was between 0.60 and 0.95, and the kappa value of the consistency test for sealant retention was between 0.73 and 0.95.

### Study outcomes

The occurrence of a new carious lesion on any surface of up to 4 treated FPMs during the 3 years of follow-up was the primary outcome for this study. The secondary outcomes were the mean number of decayed, missing, or filled permanent teeth (DMFT) and sealant retention in the FPMs. Sealant retention was classified as total, partial, or zero retention. The sealant was considered partially lost if it did not cover all occlusal pits and fissures [17].

### Statistical analysis

Baseline characteristics were compared between the sealant group and the control group. The chi-square test was used to evaluate the distributions of discrete variables. The cumulative incidence of caries and the mean number of DMFT in the FPMs were calculated according to sex and the district area for each cohort. We used Cox proportional hazard regression models for the primary outcome (e.g., the cumulative incidence of caries) and mixed linear regression (e.g, the number of DMFT) to estimate the adjusted risk of dental caries in the FPMs in the sealant group compared with that in the control group, and adjusted hazard ratios (HRs) and 95% confidence intervals (CIs) were estimated using a Cox model with adjustments for sex and district area as appropriate. To explore the anticaries effect in children treated with pit and fissure sealant, considering the effect of school as a cluster, multilevel random-effects models (individual level and school level) were developed with the mean number of DMFT as the dependent variable and treatment with pit and fissure sealant (grouped into the sealant group and the control group) as the independent variable with adjustments for sex and district area as appropriate.

All statistical analyses were performed using SPSS 21.0 software statistical software package (SPSS Inc., Chicago, IL), and *P* < 0.05 in 2-tailed tests was considered significant.

## Results

### Characteristics of the study sample

Among the eligible children, 9,298 agreed to participate in the study, although 80 children were lost to follow-up, resulting in a final sample of 9,218 children aged 6–8 years, with valid response rate of 80.7% (9,298/11,520). The characteristics of the study population are summarized in Table 1. Of the total of 9,218 children, 14,002 teeth in 4,822 children had been treated with pit and fissure sealant 3 years prior to the study, and 12,961 teeth in 4,396 children did not receive any treatment. The distributions of sex and age were similar in both cohorts (Table 1). A comparison between those who completed the assessments at the follow-up, *n* = 9,298) and those who were lost to follow-up (*n* = 2,302) revealed no major differences in children’s baseline characteristics. In the sealant group, 5,237 teeth had fully retained the sealant, 4,875 had partially retained the sealant, and 3,890 had no retention of the sealant. The sealant retention rate was 72.2%.

**Table 1:** Baseline demographic characteristics of the sealant and control groups

Variable	Sealant group	Control group	*P* value
	*N* = 4822(%)	*N* = 4396(%)	
Sex			
Female	2515(52.2)	2434(55.4)	> 0.05
Male	2307(47.8)	1962(44.6)	> 0.05
District area			
Urban	4107(85.2)	3608(82.1)	> 0.05
Rural	715(14.8)	788(17.9)	> 0.05
Total	4822	4396	

### The dental caries detection rate and the caries prevention effect 3 years after sealant treatment

From September 2011 to September 2014, 702 children with dental caries were identified for the sealant cohort, and 1,021 children with dental caries were identified for the comparison cohort. A total of 981 teeth with dental caries were detected in the sealant group, and the cumulative incidence of caries was 7.01% (981/14,002). In the control group, 1,529 teeth had dental caries, and the cumulative incidence of caries was 11.80% (1,529/12,961). The rate of dental caries detection in the FPMs in the control group was higher than that in the sealant group, and the difference was statistically significant (*P* < 0.001).

### Comparison of dental caries and the anticaries effect by sex in children treated with pit and fissure sealant 3 years previously

The cumulative incidence and relative risk of developing dental caries in the first permanent molars by sex are shown in Table 2. The cumulative incidence of caries in the control group was higher than that in the sealant group, and the difference was statistically significant regardless of sex. Children in the sealant group had a 37% decreased risk of dental caries compared with the control group (adjusted HR = 0.63 [95% CI, 0.57–0.69], *P* < 0.001). The risk of developing dental caries in the FPMs decreased by 41% in boys, reflecting a greater reduction than that in girls (34%) (male: adjusted HR = 0.59 [95% CI, 0.51–0.68]; female: adjusted HR = 0.66 [95% CI, 0.58–0.76], *P* < 0.001). The results indicated that the dental caries rate was not affected by sex (as χ^2^ = 26.23, *P* < 0.001 in the sealant group and χ^2^ = 10.19, *P* < 0.01 in the control group).

**Table 2:** Cumulative incidence and relative risk of developing dental caries in the first permanent molars by sex and area

	Sealant group			Control group			Adjusted HR^*^ (95% CI)	*P* value
	N	Case	Cumulative incidence of caries (%)	N	Case	Cumulative incidence of caries (%)		
Sex								
Male	2515	315	12.5	2434	522	21.4	0.59 (0.51–0.68)	< 0.001
Female	2307	387	16.8	1962	499	25.4	0.66 (0.58–0.76)	< 0.001
District area								
Urban	4107	581	14.1	3608	787	21.8	0.65 (0.58–0.72)	< 0.001
Rural	715	121	16.9	788	234	29.7	0.56 (0.45–0.70)	< 0.001
Total	4822	702	14.6	4396	1021	23.2	0.63 (0.57–0.69)	< 0.001

*FPMs* = first permanent molars, *HR* = hazard ratio, *CI* = confidence interval. * using Cox model, adjusting for children sex and district area appropriately

The mean number of DMFT among the FPMs and difference between the sealant group and control group by sex are shown in Table 3. The mean number of DMFT in the control group was higher than that in the sealant group, and the mean difference was statistically significant regardless of sex.

**Table 3:** Mean DMFT in the FPMs and difference between sealant group and control group by sex and area

	Sealant group			Control group			Mean difference* (95%CI)	*P* value
	N	DMFT	DMFT Mean ± SD	N	DMFT	DMFT Mean ± SD		
Sex								
Male	2515	431	0.17 ± 0.51^#^	2434	786	0.32 ± 0.71	−0.15(−0.19 to −0.12)	< 0.001
Female	2307	550	0.24 ± 0.61^#^	1962	743	0.38 ± 0.75	−0.14(− 0.18 to − 0.10)	< 0.001
District area								
Urban	4107	821	0.20 ± 0.56^#^	3608	1194	0.33 ± 0.72	−0.13(− 0.16 to − 0.10)	< 0.001
Rural	715	160	0.22 ± .56^#^	788	335	0.43 ± 0.76	−0.20(− 0.27 to − 0.13)	< 0.001
Total	4822	981	0.20 ± 0.56	4396	1529	0.35 ± 0.73	−0.14(− 0.17 to − 0.12)	< 0.001

*FPMs* = first permanent molars, *CI* = confidence interval. * using mixed linear regression analysis, adjusting for children sex and district area appropriately. ^#^ compare the difference of mean number DMFT in the FPMs between the sealant group and control group, *p*< 0.05

### Comparison of dental caries and the anticaries effect of pit and fissure sealant treatment 3 years previously between children in urban and rural areas

Table 2 shows the cumulative incidence and relative risk of developing dental caries in the FPMs by urban versus rural areas. The results show that in both urban and rural areas, the cumulative incidence of caries in children who did not receive pit and fissure sealant was higher than that in the sealant group, and the difference was statistically significant. Children in the sealant group had a lower risk of dental caries compared with children in the control group (urban: adjusted HR = 0.65 [95% CI, 0.58–0.72]; rural: adjusted HR = 0.56 [95% CI, 0.45–0.70], *P* < 0.001). The risk of developing dental caries in the FPMs in the rural students decreased by 44% 3 years after pit and fissure sealant treatment, which was higher than the rate among the urban children (35%).

The mean number of DMFT among the FPMs and the difference between the sealant group and control group by district area are shown in Table 3. The mean number of DMFT in the control group was higher than that in the sealant group, and the mean difference was statistically significant regardless of district area.

### Comparison of dental caries and the caries prevention effects among different tooth sites

The cumulative incidence of developing dental caries in the FPMs among the maxillary and mandibular teeth is shown in Table 4. The number of caries lesions in the sealant group was significantly lower than that in the control group for both the upper and lower teeth (*P* < 0.001). In addition, the cumulative incidence of caries in the mandibular teeth was significantly higher than that in the maxillary teeth during the same period (the difference in the caries rate for the maxillary and mandibular teeth in the sealant group was χ^2^ = 178.51, *P* < 0.001; the difference in the caries rate for the maxillary and mandibular teeth in the control group was χ^2^ = 292.79, *P* < 0.001).

**Table 4:** Cumulative incidence of developing dental caries in the maxillary and mandibular teeth of the FPMs

Tooth site	Sealant group			Control group			χ^2^ value	*P* value
	All the FPMs	Dental caries	cumulative incidence of caries (%)	All the FPMs	Dental caries	cumulative incidence of caries (%)		
Maxilla	12234	821	6.71*	10917	1194	10.94*	129.68	< 0.001
Mandible	1768	160	9.05	2044	335	16.39	45.20	< 0.001
Total	14002	981	7.01	12961	1529	11.80	182.98	< 0.001

*FPMs* = first permanent molars; * compare cumulative incidence of caries between maxilla and mandible, *P* < 0.001

## Discussion

Dental caries is the most significant oral disease among children, and the prevention and control of dental caries constitute a primary public health issue in China [18]. Pit and fissure sealant has been recommended to prevent caries for more than 50 years [19–21], and a project to prevent caries in the FPMs by applying pit and fissure sealant has been carried out in central and western Chinese cities since 2008 [22]. As one of the most representative south Chinese megacities, Guangzhou initiated a city-wide pit and fissure sealant program in 2011.

Sealant retention and the cumulative incidence of caries are two indicators of the long-term anticaries effects of pit and fissure sealant [23]. Our cohort study indicated that the rate of sealant retention in the FPMs was 72.2%. Children in the sealant group had a 37% decreased risk of dental caries compared with children in the control group (adjusted HR = 0.63 [95% CI, 0.57–0.69], *P* < 0.001). Compared to no use of sealant, the use of pit and fissure sealants reduced the risk of developing dental caries by 44% after 3 years in the FPMs of children from rural areas, reflecting a greater reduction than that among urban children (35%) during the same period (urban: adjusted HR = 0.65 [95% CI, 0.58–0.72]; rural: adjusted HR = 0.56 [95% CI, 0.45–0.70], *P* < 0.001). The mean number of DMFT in the control group was higher than that in the sealant group, and the difference was statistically significant regardless of sex. The caries incidence was lower for treated FPMs in both the maxilla and mandible than that for untreated teeth among both boys and girls in both urban and rural areas. The retention rate of sealant was 72.2% in Guangzhou children after 3 years, which is higher than the rates reported for Beijing (61.6%) and in the foreign literature (58%) [24].

In this study, children in the sealant group had a 37% decreased risk of dental caries in the FPMs compared with children in the control group after 3 years, indicating that pit and fissure sealant is effective for preventing dental caries. The proportion of the decreased risk of developing dental caries in the FPMs was higher than that reported for Beijing (36.4%) but lower than that in foreign reports (65%) 3 years after treatment [24–27].

The cumulative incidence of caries and the mean number of DMFT among the FPMs in the rural children in the sealant and control groups were higher than those for the urban children. The cumulative incidence of caries was 29.7% among the rural children in the control group. Among the children who had indications for pit and fissure sealant but did not undergo the treatment, 1.5 of 5 teeth decayed in 3 years. This study also found that compared with the non-use of sealants, with the use of pit and fissure sealants, the proportion of the decreased risk of developing dental caries in the FPMs among the rural children was higher than that among the urban children, and the difference was significant, indicating that the pit and fissure sealant project is superior for preventing dental caries in rural children versus urban children.

The results of the epidemiological investigation in Guangzhou in 2008 showed that the caries prevalence among rural children aged 6, 9 and 12 years was higher than that among urban children [12]. This phenomenon did not occur in Beijing, Shanghai or other areas of Guangdong, suggesting that the rural children in Guangzhou should be a focus group for preventing caries [28]. To prevent and control children’s dental caries in the FPMs in rural areas, the contents of trace elements, dental plaque, diet structure, lifestyle and oral hygiene habits in rural areas should be extensively investigated and analyzed.

The study also observed that mandibular teeth had a higher cumulative incidence of developing dental caries than maxillary teeth, and this finding may be related to the quality and retention of sealant. Furthermore, individual caries may differ in susceptibility because of differences in diet structure, habits and lifestyle, indicating that dental caries is affected not only by the pit and fissure sealant but also by other relative factors [29, 30].

The strengths of our study include the large representative sample of children and the use of longitudinal, population-based data. This study covered 12 districts of Guangzhou City and included nearly 9,300 children, and more than 26,000 FPMs were examined, yielding the largest sample available for evaluating the effects of pit and fissure sealant and thus uncovering potential prevention implications. One limitation of this study was the lack of information on relevant variables such as diet, behaviors, lifestyle and socio-economic status; therefore, we could not adjust for such potential confounders in the analysis, and residual confounding may be present. In addition, cohort studies such as the present investigation are more susceptible to selection bias than randomized controlled trials and therefore have a lower level of evidence of causation.

## Conclusions

In conclusion, compared with group without sealants, our results demonstrated that the use of sealants 3 years after treatment reduces the incidence of carious lesions in the FPMs by 44% in children from rural areas, reflecting a greater reduction than that among urban children (35%) during the same period. However, the long-term anticaries effects of pit and fissure sealant requires more observation time.

## Data Availability

Data sharing: Participant-level data are available from the corresponding author.

## Abbreviations

CI: Confidence interval
CPI: Community periodontal index
FPMs: First permanent molars
HR: Hazard ratio
